# The unequal variance signal-detection model of recognition memory: Investigating the encoding variability hypothesis

**DOI:** 10.1177/1747021820906117

**Published:** 2020-02-27

**Authors:** Rory W Spanton, Christopher J Berry

**Affiliations:** School of Psychology, University of Plymouth, Plymouth, UK

**Keywords:** Recognition memory, signal-detection theory, encoding variability, unequal variance, old item variance, memory strength

## Abstract

Despite the unequal variance signal-detection (UVSD) model’s prominence as a model of recognition memory, a psychological explanation for the unequal variance assumption has yet to be verified. According to the encoding variability hypothesis, old item memory strength variance (σ_o_) is greater than that of new items because items are incremented by variable, rather than fixed, amounts of strength at encoding. Conditions that increase encoding variability should therefore result in greater estimates of σ_o_. We conducted three experiments to test this prediction. In Experiment 1, encoding variability was manipulated by presenting items for a fixed or variable (normally distributed) duration at study. In Experiment 2, we used an attentional manipulation whereby participants studied items while performing an auditory one-back task in which distractors were presented at fixed or variable intervals. In Experiment 3, participants studied stimuli with either high or low variance in word frequency. Across experiments, estimates of σ_o_ were unaffected by our attempts to manipulate encoding variability, even though the manipulations weakly affected subsequent recognition. Instead, estimates of σ_o_ tended to be positively correlated with estimates of the mean difference in strength between new and studied items (*d*), as might be expected if σ_o_ generally scales with *d*. Our results show that it is surprisingly hard to successfully manipulate encoding variability, and they provide a signpost for others seeking to test the encoding variability hypothesis.

Extensive research has focused on applying signal-detection theory to recognition memory (see Rotello, 2017, for a review)—the ability to judge whether or not an item (e.g., a word) has been encountered before in a particular context. Since the first attempts to model recognition memory, the unequal variance signal-detection model (UVSD) has been accepted as one of the most successful formal models. In the UVSD model, recognition judgements are modelled as arising from a unidimensional memory strength variable. The strength of old (studied) or new (non-studied) items are represented as two separate normal (Gaussian) distributions, with the mean of the old item distribution (µ_o_) being typically greater than that of the new item distribution (µ_n_: typically fixed at 0). The difference between the old and new item distribution means is henceforth referred to as *d*. Recognition confidence ratings can be modelled by comparing an item’s strength value to criteria values at various intervals of memory strength. Each criterion represents a level of confidence in a recognition judgement, ranging from a high confidence that an item was new (resulting from low memory strength) to a high confidence that an item was old (resulting from high strength).

The UVSD model’s success has been consistently reflected in accurate predictions of patterns in observed data. A common analysis of recognition data is the creation of a receiver operating characteristic (ROC), which is a plot of the hit rate (proportion of correctly recognised old items) against the false-alarm rate (the proportion of new items incorrectly recognised) at different levels of the response criterion. The UVSD model can account for several established regularities in observed ROCs (Yonelinas & Parks, 2007). It also predicts a linear *z*-transformed ROC (*z-*ROC), which is often seen in item recognition studies. Previous studies have shown that the slope of the *z-*ROC is commonly close to 0.8 (Glanzer et al., 1999; Ratcliff et al., 1992). As the value of the *z-*ROC slope represents the ratio of new to old item variance in the UVSD model, this shows that the variance of the old item strength distribution is approximately 1.25 times that of the new item distribution. The UVSD model accounts for this *old item variance effect* by allowing the standard deviation of the old item strength distribution (σ_o_) to be a free parameter, which can be greater than the standard deviation of the new item strength distribution (σ_n_: typically set at 1). Thus, both the strength and variance of the old item distribution can scale relative to the new item distribution. With the inclusion of σ_o_, the UVSD model can be expressed as having parameters θ *=* {*c*_1_, *c*_2_, . . ., *c*_I_, *d*, σ_o_}, where *c*_I_ represents the highest decision criterion in terms of associated strength (Kellen et al., 2013). The probability of a “hit” response (a correct “old” judgement) can be expressed as

(1) $$P\left(H\right)=\Phi \left(\frac{d-c_{i}}{\sigma_{o}}\right)$$

where Φ is the cumulative normal distribution function, and *c_i_* is a given strength criterion. The probability of a false alarm (an old item being incorrectly judged as “new”) is

(2) $$P\left(FA\right)=\Phi \left(-c_{i}\right)$$

## The encoding variability account

One psychological explanation that has been put forward to explain why the variance of the old item distribution is greater than that of the new item distribution in the UVSD model is the encoding variability hypothesis (Jang et al., 2012; Wixted, 2007). This is the idea that the strength of each old item is incremented by a variable, rather than a fixed, amount of strength at study (Wixted, 2007). Formally, the memory strength of an old item is the result of adding two Gaussian random variables, representing a baseline strength for all items and additional strength for old items, respectively (Jang et al., 2012). Using this definition, additional strength is assumed to be the result of psychological variables that affect memory strength at encoding (henceforth, *encoding variables*). Examples of encoding variables could presumably include the duration for which a participant studies a stimulus, the amount of attention paid to a stimulus, or some other form of stimulus–participant interaction. As it is likely that the effect of these variables would vary from trial-to-trial, the UVSD model is arguably more plausible than an equal variance signal-detection model, which would explain the effect of these variables as being fixed (Wixted, 2007). The total memory strength of an old item can therefore be expressed as *O* *=* *B* *+* *Y* where *B* is the baseline memory strength of an item, and *Y* is strength added as a result of encoding variability. Both *B* and *Y* are assumed to be normally distributed random variables that are independent of each other, so that

(3) $$B\sim N\left(\mu_{baseline},\sigma_{baseline}\right)$$

(4) $$Y\sim N\left(\mu_{added},\sigma_{added}\right)$$

It is also important to note that this is only one explanation for the unequal variance assumption—there is nothing inherent in the specification of the UVSD model that compels this particular account, and a failure to support the hypothesis should not be equated with a failure to support the UVSD model.

## The recollection account

Although our focus in this article is on the encoding variability account, we also give consideration to two other prominent models of recognition and their accounts of the old item variance effect. According to the dual process signal-detection (DPSD) model (Yonelinas, 1994), the old item variance effect arises because two independent memory processes, recollection and familiarity, drive recognition. When an item is presented at test, it has a chance of being recollected as a studied item if the memory strength associated with it is greater than a certain threshold. This is expressed parametrically as *R*, the probability that a studied item will be recollected, and as a result judged old with the highest degree of confidence. If an item does not surpass this threshold, the recognition judgement is determined by familiarity, an equal variance signal-detection process (i.e., where σ_o_ = σ_n_ = 1). Familiarity represents cases where a stimulus seems familiar, but in the absence of remembering contextual details (Mandler, 1980). Despite this, “familiar” items can still receive the highest recognition confidence rating in the same way that any old item could in an equal variance signal-detection model. Because of the equal variance assumption, the mean difference between the new and old item distributions of familiarity (i.e., µ_o_) is equivalent to *d′*, and represents memory strength within the familiarity process. The DPSD model has parameters θ *=* {*c*_1_, *c*_2_, . . ., *c*_I_, *d′, R*} (Kellen et al., 2013); the probability of a false-alarm response is expressed in Equation 2, and the probability of a hit response is defined as

(5) $$P\left(H\right)=R+\left(1-R\right)\Phi \left(d\prime -c_{i}\right)$$

A relative increase in *R* results in a greater number of old items having high amounts of memory strength associated with them, and this would increase the variance (and mean) of the old item strength distribution relative to the new item distribution. Therefore, the adjustment of *R* can account for changes in old item variance. When the DPSD model was first conceptualised, Yonelinas (1994) described recollection as an “all or none” process; this has since been interpreted to suggest that recollected items are homogeneous in strength (Wixted, 2007). Parks and Yonelinas (2007) later clarified that recollected items are graded in memory strength. In further clarification, Koen and Yonelinas (2010) stated that the recollected and familiar item distributions do not overlap. However, the strict distributional assumption for recollected items remains unspecified; although commonly depicted as square, the distribution could take on any shape (Yonelinas et al., 2010). Without this information, it is not possible to determine a theoretical value of *R* that maximises old item variance without making several assumptions upon limited supporting evidence. Although this is not a major issue as one can still assume that *R* increases to an unknown value to account for greater levels of old item variance, this limitation makes it more difficult to determine a precise relationship between old item variance and the value of *R*. As the distribution of old item strength in the DPSD model can be conceptualised as a mixture of the recollection and familiarity distributions, it can also be assumed that, given a fixed value of *R*, a lower *d′* would also lead to greater old item variance, because this would increase the distance between the two distributions. Again, in the absence of clearly defined characteristics of the distribution of recollected items, the extent of this effect is unknown. However, it is certain that both *d′* and *R* help to determine both the mean and variance of old item strength.

## The mixture account

A third prominent account of the old item variance effect is offered by the mixture signal-detection (MSD) model (DeCarlo, 2002). Like the DPSD model, recognition of new items is solely derived from a single distribution in this model. Old items are represented by multiple Gaussian distributions (unlike in the UVSD and DPSD models), which correspond to different levels of processing that items receive during encoding. A common example is that some items may be fully attended to during study, whereas others are only partially attended to. In the case where an item is only partially attended to, it would fall into a distribution of partially attended old items (A′). If an item is fully attended to, it is represented in a separate distribution of fully attended old items (A). Although A′ could have a greater mean strength value than the distribution of new items (N), this would still be less than the mean of A, because the items in A were encoded more strongly due to them being attended to at a higher level. The difference between the mean value of A′ and N is defined as *d*_A′_, which provides a measure of the comparative strength of the two distributions. This value can also affect key assumptions made by the model. For example, if *d*_A′_ = 0, this implies that items in A′ are not attended to at all in the study phase, since µ_A′_ *=* µ_N_ (DeCarlo, 2002). This contrasts with higher values of *d*_A′_, which assumes that the A′ distribution still received a notable increment in strength in comparison with new items. Despite this, DeCarlo (2002) found that assuming *d*_A′_ = 0 yields non-significant values of *G*^2^ and likelihood ratio test statistics when the MSD model is fitted to data with this assumption. This provides evidence that estimates from this constrained MSD model fitted the data adequately, suggesting that this parameter can be fixed.

In the MSD model, the parameter λ represents the proportion of trials in which an old item was fully attended to in the study phase. With this parameter, the MSD model’s parameters can be defined as θ *=* {*c*_1_, *c*_2_, . . ., *c*_I_, *d*_A_, *d*_A′_, λ} (Kellen et al., 2013); the probability of a false alarm is expressed in Equation 2, and the probability of a hit response can be formally described as

(6) $$P\left(H\right)=\lambda \Phi \left(d_{A}-c_{i}\right)+\left(1-\lambda \right)\Phi \left(d_{A^{\prime }}-c_{i}\right)$$

If λ = 1, then no items are assigned to the A′ distribution; conversely, if λ = 0, no items would remain in A, and all would be assigned to the less attended A′ distribution. As the variance of each distribution in the model is equal, the model is equivalent to a traditional equal variance signal-detection model in either case where all studied items fall into one distribution (DeCarlo, 2002). This also means that, for a given difference between A′ and A, the value of λ that produces the maximum amount of old item variance is 0.5, as this reflects the largest spread of items across A and A′. Given that the difference between the N and A distributions (*d*_A_) and *d*_A′_ also represent the relative strength of each old item distribution, the variance of the old item mixture distribution can also be influenced by these parameters. Therefore, the MSD model accounts for both memory strength and old item variance through a combination of adjustments to λ, *d*_A_, and *d*_A′_. For the purposes of this article, we will assume a fixed value of *d*_A′_ = 0 to focus on changes in λ and *d*_A_ in the MSD model. This constraint eliminates a free parameter (*d*_A′_) from the MSD model, bringing the number of free parameters in line with the UVSD and DPSD models. In addition, it simplifies the interaction of the model’s parameters in their contribution to overall strength and old item variance, while still providing good fits to data (DeCarlo, 2002).

## Testing the accounts

In an attempt to test the encoding variability and recollection accounts of the old item variance effect, Koen and Yonelinas (2010) manipulated the duration for which old items were presented in the study phase. They did so by comparing a pure study condition, where items were presented for 2.5 s, to a mixed study condition where items were presented for either 1 or 4 s each. At test, participants gave responses on a 1 to 6 confidence rating scale and then made *remember, know*, or *new* judgements (Gardiner, 1988; Tulving, 1985), which were then analysed for effects of encoding variability and recollection/familiarity, respectively. Specifically, there was no difference in estimates of σ_o_ between each condition, and after subtracting estimates of recollection, the average *z-*ROC slope in both conditions did not significantly differ from 1. Therefore, Koen and Yonelinas (2010) concluded that encoding variability did not contribute to the old item variance effect and their results were instead consistent with a dual process account.

This conclusion has been contested on methodological grounds (Jang et al., 2012; Starns et al., 2012). A criticism made by both Starns et al. (2012) and Jang et al. (2012) was that Koen and Yonelinas’s (2010) method did not actually have any relevance to the encoding variability hypothesis because the effect of presenting items for 1 and 4 s in one list at test was to create a mixture distribution of old item strength. That is, this manipulation creates a separate distribution for both exposure durations used, and the underlying old item distribution is a mixture of these distributions, which is not Gaussian in form. In contrast, the encoding variability account asserts that memory strength is the sum of two Gaussian distributions (representing baseline strength and quality of encoding) and retains a Gaussian form. Because of this inconsistency, Koen and Yonelinas’s (2010) results did not test the encoding variability hypothesis; instead, their results have more relevance to an MSD account. In addition, other issues such as a lack of experimental power and use of an extended range to calculate *z*-ROCs in their analysis were raised (Jang et al., 2012; Starns et al., 2012). Although Koen and Yonelinas (2013) addressed these issues in a response, they still could not conclude that encoding variability was an unsatisfactory explanation of old item variance.

Further research by Koen et al. (2013) investigated encoding variability, recollection and attentional (mixture) accounts of old item variance, focusing on retrieval manipulations to investigate the differential claims of each theory. These manipulations included speeding response times, dividing attention, reinstating the context of encoding at test, and increasing the delay between study and test phases. Estimates of the σ_o_ parameter in the UVSD model were found to be affected by these retrieval manipulations, which seems at odds with the encoding variability hypothesis. The recollection account was found to provide the most accurate predictions, with inconsistent evidence being found for the mixture account. Although this shows that old item variance can be affected at the retrieval stage, no study has to our knowledge attempted to test the predictions of these accounts by manipulating old item variance in the study phase in a manner that would be suitable for the purposes of testing the encoding variability hypothesis (Rotello, 2017). In this study, we aim to provide methodologically valid tests of the encoding variability hypothesis in three experiments. Each of these experiments was preregistered using the Open Science Framework. A full disclosure of our aims, experimental design, methods, and statistical indices for each experiment was uploaded prior to data collection for each respective experiment. Any deviations from the preregistration for each experiment were also stated and justified after each experiment was conducted.

## Experiment 1

In this experiment, we attempt to test the encoding variability hypothesis by comparing estimates of σ_o_ following two encoding conditions; one in which items will be presented for a fixed duration (the fixed condition), and one in which items will be presented for variable durations, sampled from a normal distribution (the variable condition). This manipulation was suggested by Jang et al. (2012) as a suitable means of testing the encoding variability hypothesis because it is more likely to ensure that the underlying old item strength distribution retained a Gaussian form, rather than a mixture. Previous research confirms that increasing study duration improves memory accuracy (e.g., across durations ranging from 40 to 2,250 ms in Berry et al., 2017; 1 vs. 3 s in Jacoby & Dallas, 1981; 1 vs. 10 s in Musen, 1991; 1, 3, vs. 6.5 s in Neill et al., 1990; 50–2,000 ms in von Hippel & Hawkins, 1994), therefore varying study duration within a set of old items would be expected to increase variation in strength. By making the exposure duration at study a Gaussian variable, this would seem the most likely way of manipulating encoding variability in such a way that is equivalent to adding two Gaussian distributions to create a Gaussian product (i.e., of manipulating σ_added_ in Equation 4). This avoids the theoretical issues caused by mixing two discrete exposure duration classes to create a distribution which is not Gaussian, as Koen and Yonelinas (2010) did.

Jang et al. (2012) expressed that this method could have potential issues, such as participants rehearsing the more briefly presented items in the variable condition. To mitigate this concern, study trials in both conditions will advance automatically with the same inter-trial interval (ITI; 1 s) to minimise any potential window for rehearsal. This way, any increment in memory strength gained from further rehearsing an item into the next trial would be balanced by the decrement to the memory strength of the next item (assuming that covert rehearsal even occurs at all). While the distribution of exposure durations in the variable condition will be Gaussian, they will be selected in a way that the total duration of both the variable and fixed duration study phases will be equal. As a result, the total time to encode items in either condition will be the same. There is also the issue that, although study duration is a Gaussian variable, the resulting distribution of strengths may not be Gaussian. This is because the function that relates study duration and memory accuracy is likely to be negatively accelerated, rather than linear. It is difficult to confirm the distribution of memory strength as a latent variable; however, we would at the very least expect the variance of the resultant strength distribution to be greater as a result of this manipulation. We discuss this issue, and theoretical motivations for assuming a Gaussian product distribution in further detail in our “General Discussion.”

We hypothesise that our manipulation of study duration will increase the variability in old item strength in the variable condition, relative to old item strength variance in the fixed duration condition. In accordance with the encoding variability hypothesis, we expect a greater estimate of σ_o_ in the variable condition when the UVSD model is fitted to the data. We will also fit the DPSD and MSD models to the data given their prominence, and for parity with previous research (Koen et al., 2013). As explained above, the DPSD and MSD models can account for old item variance through changes in *d′* and *R*, or *d*_A_ and λ, respectively. Accordingly, when the DPSD model is fit to the data, we would expect estimates of *R* to be higher in the variable condition than the fixed condition, along with lower estimates of *d′*. When the MSD model is fit to the data, we would expect estimates of λ to be closer to 0.5, or estimates of *d*_A_ to be higher (or a combination of both) in the variable condition than in the fixed condition. Parameter recovery simulations (see Supplemental Materials, Appendix A) confirm that it is theoretically possible for us to observe these trends in the parameter estimates, given that the UVSD model is the true generative model.

It should be made clear at the outset that, as the UVSD, DPSD, and MSD models can all account for the old item variance effect (albeit with unique parameters), they cannot be discriminated purely on this basis (see Supplemental Materials, Appendix A). This applies to any situation in which an encoding variability manipulation is successful. Each model can however, in theory, be discriminated based on goodness of fit (GOF). Although the DPSD and MSD models can affect the variance of the old item distribution, they achieve this by making the distribution non-Gaussian. If, as suggested by Jang et al. (2012), the effect of presenting items for variable durations at encoding is to produce an underlying strength distribution that is Gaussian in form, then, because the UVSD model also assumes that the underlying strength distribution is Gaussian, it seems reasonable to expect the UVSD model to provide a better quantitative fit to the data than the DPSD and MSD models. Model recovery simulations (see Supplemental Materials, Appendix B) suggest that it is theoretically possible for us to identify the true generative model from comparisons of the fit of the UVSD, DPSD, and MSD models with *G*^2^.

### Method

#### Participants

Forty participants (six males) with a mean age of 20.78 years (*SD* = 3.41) from a University of Plymouth Participation Pool took part in this experiment. This sample size was chosen (in this experiment and the next) to provide 80% power to detect a medium-sized effect (i.e., Cohen’s *d*_z_ = 0.46) in a repeated-measures design with two levels (i.e., in a paired-samples *t* test). Each participant was fluent in English and received course credits in return for participation. One participant was excluded from the analysis for providing outlying results; their hit rates were very low (0.02 in the fixed condition, 0.12 in the variable condition) and false-alarm rates very high (0.88 in the fixed condition, 0.90 in the variable condition). This participant consistently judged new items as old and old items as new, indicating that they misunderstood the confidence rating scale. Their data were replaced with that of a new participant who completed the same counterbalancing condition, to retain the initially planned sample size and achieve even counterbalancing. All analyses were performed after this replacement was completed.

#### Materials

The stimuli were 520 seven-letter nouns. Each word had a frequency of between 1 and 30 occurrences per million (*M* = 5.73, *SD* = 6.45; Kučera & Francis, 1967). These word types and frequencies were chosen to match those used by Koen and Yonelinas (2010) to enable a comparison with their method. Participants viewed the stimuli on Viglen computers running a custom MATLAB program^1^ using the Cogent 2000 toolbox. They were presented in 40-pt Courier New font. Each stimulus in the fixed duration condition was presented for 3,000 ms. The exposure duration for each stimulus in the variable duration condition was randomly sampled from a normal distribution with a mean of 3,000 ms and standard deviation of 1,100 ms. The durations were sampled with the following constraints: (1) the minimum and maximum duration was 500 and 5,500 ms, respectively; (2) the sum of the durations equalled the sum of the durations in the fixed condition (i.e., 3,000 ms × 130 trials = 390 s), which ensured that both study phase conditions lasted for the same length of time; and (3) the sample distribution did not significantly deviate from a normal distribution, as indicated by Kolmogorov–Smirnov, *D*(129) = 0.03, *p* = .97, and Anderson–Darling, *A*(129) = 0.18, *p* = .92, tests. The upper and lower bounds were chosen to allow as much variance as possible across the sampled distribution (*SD* = 1,191 ms), while (1) mitigating participant fatigue as a result of a longer study phase, (2) ensuring that the duration was long enough for identification to occur. One set of exposure durations was generated for the variable duration condition; this set was used for all participants (see Supplemental Materials).

#### Procedure

Each participant completed both experimental conditions sequentially, in a within-subjects design. The order of the conditions was counterbalanced, such that half of the sample completed the fixed duration condition first, and the other half the variable duration condition first. The two sets of stimuli used in either condition were also counterbalanced across participants. Half of the participants viewed set 1 in their first condition and set 2 in their second condition; this order was reversed for the other half of the sample. This created a 2 (order) × 2 (stimulus set) counterbalancing design, with an equal number of participants assigned to each of the four possible counterbalancing conditions.

After providing informed consent, participants received instructions for the study phase. They were told that they would see a series of words, and that it was critical that they pay attention to each word for the full duration of its exposure. They were also told to try to memorise as many of the words as possible. In each study trial (130 trials in total), a fixation point appeared for 500 ms, after which a stimulus was presented. In the fixed duration condition, the stimulus was presented for 3,000 ms. In the variable duration condition, the stimulus was presented for a duration randomly sampled (without replacement) from the set of exposure durations. A blank inter-trial interval screen followed each stimulus presentation, lasting for 1,000 ms. After each study phase, a 3-min retention interval followed, in which participants completed word fragments corresponding to countries of the world.

The test phase followed, in which participants were shown the 130 stimuli they saw during the previous study phase, randomly intermixed with 130 new stimuli. Participants were instructed to respond to each stimulus based on their confidence that the stimulus was new or old (using the scale “1 = *sure new*, 2 = *probably new*, 3 = *guess new*, 4 = *guess old*, 5 = *probably old*, 6 = *sure old*”). Participants were instructed to prioritise the accuracy of their decision making over the speed of their response and to use all confidence ratings. In each trial, the stimulus was presented after a fixation point (again shown for 500 ms) until a response was made. A static cue was displayed throughout each trial, which reiterated the question (“New or Old?”) and each confidence level on the rating scale. After a response was made, a blank inter-trial interval screen was presented for 500 ms before the next trial began.

### Results

All analyses in this article were performed using the R statistical computing language (Version 3.4.1; R Core Team, 2017). Bayesian statistics were calculated using the BayesFactor package by Rouder et al. (2009). All of the Bayes Factors we report are scaled JZS Bayes Factors in favour of the alternative (i.e., BF_10_). A detailed explanation of our model fitting procedure, including parameter constraints, and aggregate ROCs and *z-*ROCs for each experiment, are available in our Supplemental Materials (in Appendices C and D, respectively).

#### Recognition performance

The mean hit rate and false-alarm rate across participants are shown in Table 1. A 2 × 2 within-subjects ANOVA with response (hit rate, false-alarm rate) and condition (fixed, variable) as factors revealed a significant main effect of response, *F*(1, 39) = 118.58, *p* < .001, $\eta_{p}^{2}$ = .75, BF = 1.22 × 10^30^, no significant main effect of condition, *F*(1, 39) = 2.83, *p* = .10, $\eta_{p}^{2}$ = .07, BF = 0.21, or significant interaction, *F*(1, 39) < 1, *p* = .83, $\eta_{p}^{2}$ = .001, BF = 0.27. This indicated that participants were able to successfully discriminate old from new items, and that levels of discriminability did not reliably differ between conditions.^2^

**Table 1.** Mean hit and false-alarm rates (*SE* in parentheses) for the fixed and variable conditions in Experiments 1, 2, and 3.

Experiment and condition	Hit rate	False-alarm rate
Experiment 1		
Fixed	0.65 (0.02)	0.32 (0.03)
Variable	0.63 (0.02)	0.30 (0.02)
Experiment 2		
Fixed	0.61 (0.02)	0.28 (0.02)
Variable	0.56 (0.02)	0.26 (0.02)
Experiment 3		
Low variance	0.64 (0.03)	0.31 (0.03)
High variance	0.62 (0.03)	0.34 (0.03)

#### Parameter estimates

The parameters of the UVSD, DPSD, and MSD models were estimated for each participant using maximum likelihood estimation (Dunn, 2010); this procedure was used for all model fitting procedures in this article. The mean estimates are shown in Table 2. For the UVSD model, contrary to what might be expected according to the encoding variability hypothesis, there was no significant difference between the mean estimates of σ_o_ between conditions,^3^ *t*(39) = −0.73, *p* = .47, *d* = 0.14, 95% confidence interval (CI) [−0.09, 0.20], BF = 0.22. There was also no significant difference between the mean estimates of *d* between the fixed and variable conditions, *t*(39) = 0.53, *p* = .60, 95% CI [−0.21, 0.36], BF = 0.19. If anything, the mean estimates of σ_o_ and *d* across participants were numerically greater in the fixed condition.

**Table 2.** Means and standard deviations of parameter estimates in model fits to individual data in Experiment 1.

Model	Parameter	Fixed condition		Variable condition	
UVSD	σ_o_	1.47	(0.41)	1.42	(0.36)
	*d*	1.27	(1.06)	1.19	(0.92)
	*C*_1_	−1.21	(1.04)	−1.02	(0.78)
	*C*_2_	−0.12	(0.65)	−0.04	(0.64)
	*C*_3_	0.54	(0.52)	0.62	(0.50)
	*C*_4_	1.08	(0.56)	1.13	(0.55)
	*C*_5_	1.92	(1.06)	2.00	(1.43)
DPSD	*R*	0.26	(0.22)	0.25	(0.19)
	*d′*	0.56	(0.49)	0.58	(0.48)
	*C*_1_	−1.14	(0.96)	−0.97	(0.77)
	*C*_2_	−0.12	(0.62)	−0.05	(0.63)
	*C*_3_	0.50	(0.50)	0.58	(0.48)
	*C*_4_	1.03	(0.53)	1.09	(0.53)
	*C*_5_	2.59	(1.99)	2.39	(1.94)
MSD	λ	0.58	(0.30)	0.61	(0.29)
	*d*_A_	2.60	(1.99)	2.38	(1.78)
	*C*_1_	−1.25	(1.27)	−1.01	(0.77)
	*C*_2_	−0.14	(0.64)	−0.05	(0.65)
	*C*_3_	0.52	(0.52)	0.60	(0.51)
	*C*_4_	1.08	(0.56)	1.12	(0.54)
	*C*_5_	2.20	(1.53)	2.05	(1.55)

UVSD: unequal variance signal-detection; DPSD: dual process signal-detection; MSD: mixture signal-detection.

With regard to the DPSD model, the mean estimate of *R* did not significantly differ between conditions, *t*(39) = 0.42, *p* = .68, 95% CI [−0.06, 0.08], BF = 0.19, nor did the mean estimates of *d′, t*(39) = 0.39, *p* = .70, 95% CI [−0.15, 0.10], BF = 0.18. For the MSD model, the mean estimate of λ did not differ between conditions, *t*(39) = −0.53, *p* = .60, 95% CI [−0.15, 0.09], BF = 0.19. The estimates of *d*_A_ were extremely high (greater than 10) for seven participants—four had extreme *d*_A_ estimates in the variable condition and three had extreme estimates in the fixed condition. These participants were excluded listwise when calculating the mean values of *d*_A_ (in Table 2). With these participants excluded, there was no significant difference between the mean estimates of *d*_A_ in the fixed and variable conditions, *t*(32) = 0.12, *p* = .90, 95% CI [−0.87, 0.98], BF = 0.19, nor was there a significant difference between conditions when these outliers were included (according to a Wilcoxon Signed Ranks test, *V* = 437, *p* = .72).

#### Comparisons of fit

GOF tests were performed upon model fits to each individual participant’s data, as well as aggregated data across the sample. *G*^2^ was used to assess GOF in model fits to individual and aggregated data. When participant-level model fits were assessed, the UVSD model was the best fitting model for the majority of participants in the fixed duration condition, followed by the MSD and DPSD models (see Table 3). In the variable duration condition, the DPSD model was the best fitting model for the greatest proportion of participants; the UVSD model had the second largest proportion, and the MSD model, the third. In the case of the aggregate fits, the MSD model fit the data best in both conditions; the UVSD model provided the second best fit, surpassing the DPSD model (see Table 4). All model fits to aggregated data from the variable condition were rejected on the basis of a 95% significance level; the DPSD model fit to aggregated fixed condition data was also rejected. It is worth noting that these rejections are likely due to the distortion of patterns found in individual data as a result of aggregation; therefore, the validity of each model should not be doubted purely on this basis. Regardless, the results of the model comparison are mixed and do not clearly allow the models to be discriminated.

**Table 3.** Goodness of model fits to individual participant’s data in Experiment 1, assessed by *G*^2^.

Condition	Model	Sum of *G*^2^	Percentage of best fits	Percentage of rejected fits
Fixed				
	UVSD	174.34	40	7.5
	DPSD	182.98	25	10
	MSD	155.64	35	7.5
Variable				
	UVSD	177.59	37.5	10
	DPSD	181.20	42.5	12.5
	MSD	166.06	20	2.5

UVSD: unequal variance signal-detection model; DPSD: dual process signal-detection model; MSD: mixture signal-detection.

Fits rejected if *p* < .05.

**Table 4.** Goodness of model fits to aggregate data in fixed and variable duration conditions (Experiment 1), assessed by *G*^2^.

Condition	Model	Order of best fit	*G* ^2^	*p* value
Fixed				
	UVSD	2	3.77	.44
	DPSD	3	49.79	<.01*
	MSD	1	0.53	.97
Variable				
	UVSD	2	9.98	.041*
	DPSD	3	28.83	<.01*
	MSD	1	9.65	.047*

UVSD: unequal variance signal-detection model; DPSD: dual process signal-detection model; MSD: mixture signal-detection.

* *p* < .05.

#### Unplanned analyses

As the results of Experiment 1 indicated no significant differences between σ_o_ in either condition, further analyses (which were not stated in our preregistration) were performed to investigate the possibility that the lack of a significant difference in parameter estimates between conditions was because exposure duration did not have any effect on recognition ratings at all. A Pearson correlation between the exposure duration of items and their subsequent recognition rating was calculated for each individual participant. The mean correlation was very weakly positive (*M* = .05, *SE* = .01), but was reliably greater than zero across participants, *t*(39) = 3.54, *p* < .01, 95% CI [0.02, 0.08], BF = 29.10. This suggests that items that were studied for longer tended to receive slightly higher confidence ratings at test, confirming that exposure duration did affect recognition, albeit very weakly.

### Discussion

The results of Experiment 1 failed to confirm the prediction of the encoding variability account: The mean estimate of σ_o_ did not reliably differ between the fixed and variable conditions; if anything, estimates of σ_o_ (and *d*) tended to be greater in the fixed condition (but not reliably so). Similarly, there was no evidence that old item variance increased in the DPSD or MSD model as a result of varied study duration. This means that manipulating study duration did not create additional encoding variability and result in greater old item variance, as hypothesised. Although the significant positive correlation between exposure duration and recognition confidence ratings indicates some relationship between these two variables, the size of the correlation showed that the effect of study duration on recognition was very weak. Indeed, it is unlikely that this effect would have had a noticeable effect on σ_o_, as we observed. Although these results do not rule out the encoding variability hypothesis, they do at least suggest that varying study duration over the range we used in the variable condition is not a suitable means of manipulating encoding variability, and they necessitate the search for other encoding variables, which may affect old item variance.

As the old item variance effect was present even when study duration was fixed (e.g., as shown by estimates of σ_o_ being greater than 1 in the UVSD model in Experiment 1), other variables must affect old item variance at study if the encoding variability hypothesis holds true. Another factor that presents a wide scope for creating encoding variability at study is the level of attention paid to each stimulus. Despite attempts to control the effects of attention in the study phase of Experiment 1, it is highly likely that participants’ attention fluctuated within each study phase (Smallwood & Schooler, 2015). This natural variation could have contributed to the old item variance effect observed in both conditions, overshadowing any effect of varying the exposure duration. Indeed, it may be that trial-to-trial variation in attention is a better proxy for encoding variability than trial-to-trial variation in exposure duration. We investigate this possibility next.

## Experiment 2

In Experiment 2, we aimed to investigate the effects of trial-to-trial variations in attention at encoding to provide a further test of the encoding variability hypothesis. A common method of inducing experimentally controlled divided attention is the *n*-back paradigm, in which a stimulus from a given trial is held in memory until a response relating to that stimulus is cued “*n*” trials later. One possible variant of this procedure involves digits being presented in sequential trials; on each trial, the participant judges whether the digit from the preceding trial was odd or even. This procedure can be modified so that the participant is instructed to make their judgement about the *n*th preceding trial, and at any point requires the participant to hold the *n*th digit in their working memory (or at least the response to it), as well as any successive digits (or responses). Thus, the *n*-back task^4^ can be used to divert attention from another concurrent task or stimulus presentation by presenting both tasks in different modalities (Barrouillet et al., 2004). For example, a stimulus could be presented visually, while each *n*-back digit could be presented auditorily. As a result, this method is able to mimic the fluctuation of attention between different modalities, as might be expected to occur in an ecologically valid situation, such as a learning episode (Kane et al., 2007).

The *n*-back task may be suitable for the purposes of testing the encoding variability hypothesis because the intervals between each digit presentation can be varied, for example, by randomly sampling the interval from a normal distribution. If the study duration of words on screen remains fixed during this manipulation, the presentation of visual and auditory *n*-back stimuli would become asynchronous, with participants having to make *n*-back responses at irregular intervals throughout the trial procedure. This would result in a fluctuation in the number of digit responses required in a set time. As this is directly related to working memory demands (Barrouillet et al., 2004), and as memory strength is related to sustained attention at encoding (DeBettencourt et al., 2018), this may result in normally distributed trial-to-trial variability in attention to the target stimulus at encoding. This in turn would result in normally distributed strength being added to the baseline strength of old items in such a condition. When comparing estimates of σ_o_ between conditions with fixed (synchronous) and variable intervals, the effect of attention at study upon old item variance can be tested; we present this test in Experiment 2. Assuming that trial-to-trial variability in attention is a suitable proxy for encoding variability, we hypothesise that estimates of σ_o_ will be greater in the variable interval condition than in the fixed condition.

### Method

#### Participants

Forty participants (four males) with a mean age of 20.55 years (*SD* = 4.02) participated in this experiment in exchange for course credit. They were recruited from a University of Plymouth Participation Pool.

#### Materials

The stimuli were 520 images, each consisting of a familiar object presented against a white background (taken from Zago et al., 2005, and the Bank of Standardised Stimuli, Brodeur et al., 2010). Each image was de-saturated, resized to 256 × 256 pixels, and presented on Viglen computers using a MATLAB program. Audio clips of a female computer-generated voice speaking the digits 1 to 9 were used in the study phases of each condition. In the fixed interval condition, the interval between these digit presentations was 3,500 ms, the same duration of a complete trial (i.e., 2,500 ms object presentation, 500 ms ITI and 500 ms fixation); this meant that each digit presentation was synchronised with the onset of a new object. In the variable interval condition, the intervals between the onset of each digit presentation were randomly selected from a normal distribution with a mean of 3,500 ms (*SD* = 1,100 ms) with the constraints that (1) the minimum and maximum values were 1,000 and 6,000 ms, respectively; (2) the sum of the values in the distribution were equal to the total length of the study phase (i.e., 3.5 s × 130 trials = 455 s); and (3) the distribution did not significantly deviate from a normal, *D*(129) = 0.03, *p* = .99; *A*(129) = 0.18, *p* = .91. The same sample of interval durations was used for all participants (see Supplemental Materials). The mean and standard deviation of the distribution of sampled intervals was 3,500 and 1,117 ms, respectively.

#### Design and procedure

Participants took part in both experimental conditions sequentially in a within-subjects design. A 2 (stimuli order) × 2 (stimulus set) counterbalancing design with equal participants in each possible counterbalancing condition was implemented, as in Experiment 1. Before each study phase, participants practised the one-back task that they would perform in the study phase, but without having to memorise objects at the same time. On each practice trial, a neutral stimulus (an outline of a white square) was presented for 2,500 ms, followed by a 500 ms ITI and a 500 ms fixation point (a “+” symbol) preceding the next trial. A fixation point appeared before the first stimulus, prior to the trial procedure starting. In the fixed condition, a digit was presented with the onset of each object. Digits in the variable condition were presented at varying intervals from each other, meaning that they were not synchronised with stimulus presentation. These intervals were randomly sampled from a normal distribution with constraints (see “Materials”). Participants were instructed to make a response with each spoken digit as to whether the preceding digit was odd or even. They made these decisions by pressing either the “Z” key (if the previous digit was odd) or the “M” key (if the previous digit was even). This response scheme was reiterated on screen as a static cue throughout the practice trials. Participants could proceed if they had made 10 consecutive correct responses. To ensure that participants understood the one-back task, if, in the first practice one-back phase, 40 trials elapsed and the participant had not made 10 consecutive correct responses, the task was re-explained to them by the experimenter before completing another 40 trials. All participants were prompted to see the experimenter if they had any questions about the task after completing the practice trials.

Each study phase trial (130 in total) had the same structure as the practice trials, except that an image of an object was presented on each trial, rather than a white outlined square. After each study phase, participants completed the same retention interval task used in Experiment 1 for 3 min. The format and structure of the trials in the test phase were also identical to those in Experiment 1, whereby participants made 1 to 6 confidence ratings in response to 130 old and 130 new items, which were randomly intermixed.

### Results

#### Task performance

The proportion of correct responses made in the *n*-back task in the study phase was calculated for each participant. The mean proportion of correct responses was significantly greater in the fixed condition (*M* = 0.94, *SD* = 0.10) than in the variable condition (*M* = 0.89, *SD* = 0.12), *t*(39) = 3.18, *p* < .01, 95% CI [0.01, 0.08], BF = 12. With regard to the recognition task, the mean hit rate and false-alarm rate across participants is shown in Table 1. A 2 × 2 within-subjects ANOVA with response (hit rate, false-alarm rate) and condition (fixed, variable) as factors revealed a significant main effect of response, *F*(1, 39) = 169.57, *p* < .001, $\eta_{p}^{2}$ = .81, BF = 2.96 × 10^36^, indicating that participants tended to successfully discriminate old from new items. There was a significant effect of condition, *F*(1, 39) = 12.06, *p* = .001, $\eta_{p}^{2}$ = .24, BF = 0.30, indicating that participants tended to have a more liberal response criterion for responding “old” in the fixed than variable condition. The Response × Condition interaction was not significant, *F*(1, 39) = 2.82, *p* = .10, $\eta_{p}^{2}$ = .07, BF = 0.31.

#### Parameter estimates

The mean estimates of the parameters from each model are shown in Table 5. In the UVSD model fits, the mean estimate of σ_o_ was significantly greater in the fixed condition than in the variable condition, *t*(39) = 2.33, *p* = .02, 95% CI [0.01, 0.19], BF = 1.89. The mean estimate of *d* was also significantly greater in the fixed condition, *t*(39) = 2.40, *p* = .02, 95% CI [0.03, 0.32], BF = 2.16. Similarly, in the DPSD model fits, *R* was significantly greater in the fixed condition than in the variable condition, *t*(39) = 2.41, *p* = .02, 95% CI [0.01, 0.09], BF = 2.20. DPSD estimates of *d′*, however, did not significantly differ between conditions, *t*(39) = 0.44, *p* = .66, 95% CI [−0.10, 0.06], BF = 0.19. When the data were fit with the MSD model, λ did not significantly differ between the fixed and variable conditions, *t*(39) = 0.15, *p* = .88, 95% CI [−0.11, 0.09], BF = 0.18. One participant’s data were excluded listwise from the analysis of the MSD model’s estimates of *d*_A_, as it contained a large outlying estimate greater than 10 (*d*_A_ = 164.93). The mean estimate of *d*_A_ in the fixed condition was greater, but not reliably so, *t*(38) = 1.78, *p* = .08, 95% CI [−0.06, 0.86], BF = 0.72. A Wilcoxon Signed Ranks test including the outlying value did, however, indicate a significant difference, *V* = 592, *p* < .01.

**Table 5.** Means and standard deviations of parameter estimates in model fits to individual data in Experiment 2.

Model	Parameter	Fixed condition		Variable condition	
UVSD	σ_o_	1.49	(0.33)	1.39	(0.25)
	*d*	1.14	(0.70)	0.97	(0.59)
	*C*_1_	−1.54	(1.42)	−1.32	(0.94)
	*C*_2_	−0.31	(1.17)	−0.17	(0.65)
	*C*_3_	0.65	(0.47)	0.72	(0.46)
	*C*_4_	1.25	(0.57)	1.26	(0.45)
	*C*_5_	1.99	(0.81)	1.95	(0.59)
DPSD	*R*	0.23	(0.14)	0.18	(0.13)
	*d′*	0.52	(0.38)	0.54	(0.31)
	*C*_1_	−1.52	(1.62)	−1.33	(1.35)
	*C*_2_	−0.35	(1.34)	−0.16	(0.61)
	*C*_3_	0.59	(0.43)	0.66	(0.41)
	*C*_4_	1.14	(0.46)	1.18	(0.40)
	*C*_5_	2.91	(2.22)	2.89	(2.15)
MSD	λ	0.53	(0.23)	0.54	(0.24)
	*d*_A_	2.46	(1.35)	2.05	(1.10)
	*C*_1_	−1.48	(1.27)	−1.33	(1.04)
	*C*_2_	−0.32	(1.09)	−0.19	(0.64)
	*C*_3_	0.63	(0.49)	0.71	(0.48)
	*C*_4_	1.27	(0.58)	1.34	(0.73)
	*C*_5_	2.10	(0.85)	2.22	(1.23)

UVSD: unequal variance signal-detection; DPSD: dual process signal-detection; MSD: mixture signal-detection.

#### Comparison of fits

GOF analyses (the same as in Experiment 1) were performed on an individual participant level (see Table 6) and an aggregate level (see Table 7). The MSD model accounted for the greatest percentage of participant level best fits in both conditions, with the UVSD and DPSD models placing successively. Similarly, for the aggregate level fits, the MSD model was found to fit best to the data in both conditions, followed by the UVSD and DPSD models, respectively. All aggregate-level model fits were rejected based on a *G*^2^ significance level of .05 in the fixed condition; the DPSD model was also rejected in the variable condition. This indicates that the MSD model fit best on a participant level; although given that most fits to aggregated data were rejected, a model comparison on this level is inconclusive.

**Table 6.** Goodness of model fits to individual participant’s data in Experiment 2, assessed by *G*^2^.

Condition	Model	Sum of *G*^2^	Percentage of best fits	Percentage of rejected fits
Fixed				
	UVSD	161.31	37.5	5
	DPSD	216.28	40	12.5
	MSD	118.54	22.5	2.5
Variable				
	UVSD	138.28	35	2.5
	DPSD	178.10	35	5
	MSD	115.89	30	5

UVSD: unequal variance signal-detection; DPSD: dual process signal-detection; MSD: mixture signal-detection.

Fits rejected if *p* < .05.

**Table 7.** Goodness of model fits to aggregate data in fixed and variable duration conditions (Experiment 2), assessed by *G*^2^.

Condition	Model	Order of best fit	*G* ^2^	*p* value
Fixed				
	UVSD	2	17.61	<.01*
	DPSD	3	56.25	<.01*
	MSD	1	10.45	.03*
Variable				
	UVSD	2	5.40	.25
	DPSD	3	70.09	<.01*
	MSD	1	0.49	.97

UVSD: unequal variance signal-detection; DPSD: dual process signal-detection; MSD: mixture signal-detection.

* *p* < .05.

#### Unplanned analyses

To test whether our attentional manipulation affected recognition within the variable condition, we conducted a one-factor (number of digits per trial at three levels; 0, 1, or 2 and 3) within-subjects ANOVA on mean confidence ratings. As there were very few cases where three distractor digits were presented in a single trial (*N* = 9 throughout the whole sample), these cases were combined into a single level with two distractor digits per trial (*M* number of trials across participants with 0 digits = 18.0, 1 digit = 94.3, 2 digits = 17.5, 3 digits = 1.0). The Greenhouse–Geisser sphericity correction method was used. The mean recognition rating to items did not differ according to the number of digits that were presented at study, *F*(1.98, 77.31) = 0.29, *p* = .77, $\eta_{p}^{2}$ = .007, BF = .01 (*M* recognition rating for words studied with 0 digits = 3.87, 1 digit = 3.94, and 2 and 3 digits = 3.90). The absence of a significant difference and a BF < 0.33 suggests that the number of distractor digits presented in each trial did not influence recognition confidence ratings as we had expected.

Given our finding that estimates of σ_o_ were greater when estimates of *d* were greater in the UVSD model, we conducted Pearson correlations between estimates of these parameters across participants to determine whether σ_o_ and *d* were also linked at the level of individual participants. In the fixed condition, there was a strong, significant, positive correlation between estimates of *d* and σ_o_, *r*(38) = .80, *p* < .01, 95% CI [0.65, 0.89]. A strong positive correlation between estimates of *d* and σ_o_ was also found in the variable condition, *r*(38) = .56, *p* < .01, 95% CI [0.30, 0.74]. We also conducted Pearson correlations between estimates of the strength/variance parameters from the DPSD and MSD model fits. For the DPSD model, the correlation between estimates of *d′* and *R* in the fixed condition was weakly positive, but not significant, *r*(38) = .20, *p* = .21, 95% CI [−0.12, 0.48]. Estimates of these parameters were significantly positively correlated in the variable condition however, *r*(38) = .44, *p* < .01, 95% CI [0.15, 0.66]. As in the MSD model parameter analyses, an outlying value was excluded listwise from this analysis. The MSD model’s *d*_A_ and λ parameters were significantly negatively correlated in the fixed condition, *r*(37) = −.53, *p* < .01, 95% CI [−0.72, −0.25]. Another significant negative correlation was found in the variable condition, *r*(37) = −.55, *p* < .01, 95% CI [−0.74, −0.28].

For comparison, we also conducted the same Pearson correlation between parameter estimates for Experiment 1. Strong positive correlations were found between *d* and σ_o_ in both the fixed condition, *r*(38) = .72, *p* < .01, 95% CI [0.53, 0.85], and the variable condition, *r*(38) = .69, *p* < .01, 95% CI [0.48, 0.82]. Unlike in Experiment 2, there were also significant positive correlations between estimates of *d′* and *R* in the fixed condition, *r*(38) = .48, *p* < .01, 95% CI [0.20, 0.69], as well as in the variable condition, *r*(38) = .39, *p* = .01, 95% CI [0.09, 0.62]. Seven participants were excluded listwise from the MSD model correlation analyses for having outlying values of *d*_A_. As in Experiment 2, estimates of *d*_A_ and λ were (significantly) negatively correlated in the fixed condition, *r*(31) = −.51, *p* < .01, 95% CI [−0.73, −0.20] and in the variable condition, *r*(31) = −.54, *p* < .01, 95% CI [−0.75, −0.24]. Thus, the direction and strength of each inter-parameter correlation was largely similar in both experiments.

### Discussion

Estimates of σ_o_ were significantly greater in the fixed condition than the variable condition, contrary to what might be expected according to the encoding variability hypothesis. Estimates of *d* in the UVSD model were also significantly greater in the fixed condition, indicating that the mean memory strength for old items in this condition was also higher. In the DPSD model fits, although the *R* parameter was greater in the fixed condition, indicating higher old item variance, the model’s estimates of *d′* did not differ between conditions. As *R* affects both overall recognition strength and old item variance, an increase in this parameter with no change in *d′* implies that both memory strength and old item variance were greater in the fixed condition. The estimates of the MSD model’s parameters also showed a similar trend; although λ did not differ between conditions, *d*_A_ was marginally greater in the fixed condition (albeit not reliably so), which produces greater old item strength overall and greater old item variance. Therefore, according to all models, contrary to what might be expected under the encoding variability hypothesis, there was at least a numerical trend for both old item variance and overall levels of old item strength to be greater in the fixed than variable condition, with no evidence that old item variance was greater in the variable condition.

One explanation for why overall levels of memory strength were greater in the fixed condition could be that participants found the one-back task easier to perform in this condition. Indeed, performance in the one-back task was significantly greater in the fixed condition. If the one-back task was easier, this could have resulted in more attention being paid to the objects being presented in the fixed condition, leading to stronger encoding of items in general and therefore greater strength associated with these items at test (DeBettencourt et al., 2018).

The co-occurrence of greater overall memory strength and old item variance has also been shown in previous research in patients with hippocampal lesions (Wais et al., 2006). In a method where these patients were tested at different retention intervals, it was found that increases in memory strength over shorter retention intervals were reflected in a decrease in the slope of the *z-*ROC. As the slope of the *z-*ROC reflects the ratio of new and old item variance, an increase in memory strength can be associated with an increase in old item variance. Glanzer et al. (1999) also found that the slope of the *z-*ROC was linked to changes in recognition accuracy, again indicating that old item variance increases with overall strength. Indeed, results from Koen et al. (2013) showed the same trend; estimates of old item variance and strength in the UVSD model decreased or increased simultaneously in each of their experiments.

The notion that old item variance increases with overall strength is also supported by inter-parameter correlations from each considered model; particularly, strong positive correlations between *d* and σ_o_ in the UVSD model. As mean old item strength and variance are independently represented by *d* and σ_o_, respectively, this indicates that both are strongly linked in the UVSD model. In the DPSD model, *d*′ and *R* can affect overall strength and old item variance; increases in *d*′ increment overall strength and decrease old item variance, and increases in *R* increment both overall strength and old item variance. As positive correlations between both parameters were observed in Experiments 1 and 2 (and all but one being significant), an increase in overall memory strength can be assumed. Due to the lack of assumptions specified for the recollection distribution, it is difficult to judge how well either parameter could compensate for changes in old item variance resulting from the other. Therefore, the evidence for whether the DPSD model’s parameters showed a relationship between strength and old item variance is inconclusive. In the MSD model, *d*_A_ and λ had strong negative correlations in both conditions, demonstrating another co-occurrence of overall strength and old item variance. As λ decreases, a greater proportion of items is assigned to the A′ distribution, resulting in a reduction of total old item strength. As *d*_A_ increases, both strength and old item variance become greater. Therefore, by taking on higher values, *d*_A_ can compensate for any decreases in strength and changes in variance that occur as a result of λ having a lower value. Thus, a negative correlation between values of *d*_A_ and λ aligns with an account where strength and old item variance are related.

It is also relevant to note that the MSD model emerged as the best fitting model when GOF to individual and aggregated data was compared. Because each model was shown to be recoverable at numbers of trials equivalent to those in participant- or aggregate-level analyses of fit (see Supplemental Materials, Appendix B), the MSD model can be selected as the best fitting model across individual participants. However, as several aggregate-level fits were rejected in Experiment 2, it is difficult to advocate the superiority of any model in this case. It should also be mentioned that the mean confidence ratings did not differ according to the number of digits presented at encoding. Although this suggests that our manipulation did not affect encoding variability, it is a possibility that the number of digits per trial is not a good proxy by which to measure the effectiveness of our manipulation. Our aim in the present experiment was to vary attention continuously across the study phase in the variable condition. It is therefore difficult to measure the effectiveness of our manipulation at the level of individual trials. In this way, it is still possible that our manipulation contributed in some way to the observed effects upon old item variance and memory strength, but not according to the number of distractor digits presented per trial.

Although there is no evidence that trial-to-trial variation in attention is responsible for producing increases in old item variance consistent with the predictions of the encoding variability hypothesis, there is still a possibility that these predictions could be elicited by other variables. This could be the case if a variable with a stronger effect on encoding variability was found. Thus, the search for a method which induces encoding variability can be extended to word frequency.

## Experiment 3

Experiment 3 aims to manipulate old item variance by using word frequency as a potential encoding variable. The finding that low-frequency words (those less likely to appear within a given lexical corpus) elicit more accurate recognition judgements at test than more common words has been widely reported (Glanzer & Bowles, 1976). This applies for both types of item classes; when low-frequency words are presented as either old or new stimuli, they are more likely to be judged correctly as such. This “mirror effect” has promising implications for encoding variability. If recognition memory has a negative relationship with word frequency (Gorman, 1961), then an encoding variability account would predict that varying this according to a normal distribution would increase σ_o_ in the UVSD model. Furthermore, if the mean of this distribution were constrained to be approximately equal to that of a comparative set of words with a low variance in their frequency, the overall recognition performance for either set of words would be unlikely to differ. Thus, a “clean” test of encoding variability in which recognition strength is theoretically unlikely to differ across conditions could be achieved.

Several considerations must be made when manipulating word frequency as an encoding variable; one of the foremost is choosing an appropriate measure of word frequency. Historically, the commonality of a word was assessed by its occurrence in a corpus of one million words in total. Many researchers have used the Kučera and Francis (1967) word frequency measure, which works in this way; indeed, we adopted this metric in Experiment 1 to enable a comparison with Koen and Yonelinas’s (2010) method. However, there are problems with this measure. First, the corpus from which Kučera and Francis (1967) derived their word frequencies is unlikely to be representative of contemporary language, both due to the time in which it was selected, and its literary format. Second, the measure is unable to account for very low–frequency words (<1 frequency per million) which make up around 80% of the lexicon (Van Heuven et al., 2014).

In Experiment 3, we chose stimuli from the SUBTLEX-UK database (Van Heuven et al., 2014) and adopted the associated Zipf unit measure of word frequency. With word frequencies indexed from large, contemporary, televised British-English corpora, the SUBTLEX-UK database has a better chance of accurately representing word frequency than older measures like Kučera-Francis. In addition, the Zipf scale provides a logarithmically transformed measure of word frequency that can account for the whole lexicon on a scale of 1 to 7. From this, it is possible to sample a Gaussian distribution with a moderate frequency mean (e.g., 3.5) which, in theory, has a good chance of adding variance to memory strength.

It is also important that a set of old items chosen according to a Gaussian Zipf score distribution is accompanied at test by a closely matched new item distribution with the same constraints. If this was not the case and the new item word frequency distribution had a notably lower variance, then old words with high or low frequencies could stand out and be more identifiable in comparison. Similarly, it is important that the means of the new and old item distributions are equal, as a difference could artificially alter the overall memory strength for either stimuli set. Therefore, the use of matched old and new item Zipf score distributions ensures that task performance is controlled.

In accounting for these potential pitfalls, we present a manipulation which stands a good chance of providing a theoretically sound encoding variability effect without affecting overall memory strength. Assuming that word frequency is a suitable proxy for encoding variability, we hypothesise that estimates of σ_o_ will be greater in a high word frequency variance condition than in a low word frequency variance condition.

### Method

#### Participants

Forty participants (six males) with a mean age of 21.7 years (*SD* = 5.99) participated in this experiment in exchange for course credit. They were recruited from a University of Plymouth Participation Pool. Each participant spoke English as their first language and was non-dyslexic.

#### Materials

A total of 400 five-letter nouns from the SUBTLEX-UK (Van Heuven et al., 2014) database were used as stimuli; names and hyphenated words were excluded from consideration. Two sets of stimuli (*N* = 100 per set) were used in each item variance condition. Each set of items in the low-variance condition had Zipf unit means of 3.48, lower bounds of 3.41, and upper bounds of 3.59. These scores represent moderate word frequency (Van Heuven et al., 2014), and are the equivalent of approximately three occurrences per million words. In the high-variance condition, each set of words (H1 and H2) had a Zipf score distribution adhering to a truncated normal shape. These distributions adhered to the following constraints: (1) both distributions had a mean of 3.5 and similar standard deviations (H1: *SD* = 1; H2: *SD* = 0.99), (2) each distribution had a lower bound of 1.17 and a higher bound of either 5.83 (H1) or 5.84 (H2), and (3) were found not to significantly deviate from a normal distribution by Anderson–Darling tests (H1: *A* = 0.19, *p* = .90; H2: *A* = 0.11, *p* = .99) and Kolmogorov–Smirnov tests (H1: *D* = 0.04, *p* = .93; H2: *D* = 0.03, *p* = .99).

#### Procedure

Participants took part in both experimental conditions sequentially in a within-subjects design. A 2 (condition order) × 4 (old/new stimulus set) counterbalancing system was implemented, with an equal number of participants being assigned to each counterbalancing condition. After giving consent, participants completed either the high- or low-variance condition. In both study phases, each trial (*N* = 100) was comprised of a fixation point (a “+” symbol) presented for 500 ms, followed by a randomly selected old stimulus which was presented for 2,000 ms, and a 500 ms ITI preceding the next trial. As in Experiment 1, participants were instructed that it was important to pay sustained attention to each stimulus during the study phase. After the study phase had elapsed, participants completed the retention interval task from Experiments 1 and 2 for 3 min. The successive test phase procedure was also the same as in Experiment 1, with the sole difference being the number of stimuli presented (100 old and 100 new).

### Results

#### Recognition performance

The mean hit and false-alarm rates in both conditions are presented in Table 1. A 2 × 2 within-subjects ANOVA with response (hit rate, false-alarm rate) and condition (fixed, variable) as factors revealed a significant main effect of response, *F*(1, 39) = 109.33, *p* < .001, $\eta_{p}^{2}$ = .74, BF = 1.80 × 10^21^, indicating that participants tended to successfully discriminate old from new items. There was no significant effect of condition, *F*(1, 39) = 0.07, *p* = .79, $\eta_{p}^{2}$ = .002, BF = 0.17, nor was the Response × Condition interaction significant, *F*(1, 39) = 2.44, *p* = .13, $\eta_{p}^{2}$ = .06, BF = 0.42. Thus, the ability to discriminate old and new items in each condition was approximately equal.

#### Word Frequency effect

To gauge the degree to which our manipulation of word frequency influenced recognition confidence judgements, Pearson correlations between Zipf scores and recognition confidence judgements for old items were calculated for each participant. The mean correlation *r* value was −.10 (*SE* = 0.03); this was significantly lower than zero, *t*(39) = −3.86, *p* < .01, 95% CI [−0.15, −0.05], BF = 60.24. This suggests that lower frequency words received higher confidence ratings, although this relationship was weak.

#### Parameter estimates

All mean parameter estimates for both low- and high-variance conditions are found in Table 8. Mean estimates of σ_o_ in the UVSD model did not significantly differ between conditions, *t*(39) = −0.21, *p* = .83, 95% CI [−0.22, 0.17], BF = 0.17. Mean UVSD estimates of *d* were also not significantly different between conditions, *t*(39) = 1.20, *p* = .24, 95% CI [−0.14, 0.53], BF = 0.33. In the DPSD model, mean estimates of *R* did not differ significantly between conditions, *t*(39) = −0.13, *p* = .90, 95% CI [−0.07, 0.06], BF = 0.17, nor did mean estimates of *d*′, *t*(39) = 1.52, *p* = .14, 95% CI [−0.04, 0.26], BF = 0.49. In the MSD model, mean estimates of λ did not differ between groups, *t*(39) = −0.52, *p* = .60, 95% CI [−0.15, 0.09], BF = 0.19. Fits to four participants’ data were excluded listwise from subsequent analyses for having outlying estimates of *d*_A_ (greater than 10). *d*_A_ tended to be greater in the fixed condition, *t*(35) = 2.04, *p* = .05, 95% CI [0.003, 1.31], BF = 1.13. A Wilcoxon Signed Rank test which included the outlying estimates of *d*_A_ did not support this conclusion, however, *V* = 469, *p* = .44.

**Table 8.** Means and standard deviations of parameter estimates in model fits to individual data in Experiment 3.

Model	Parameter	Low-variance condition		High-variance condition	
UVSD	σ_o_	1.41	(0.55)	1.43	(0.56)
	*d*	1.18	(1.16)	0.98	(0.81)
	*C*_1_	−1.09	(1.05)	−1.08	(1.19)
	*C*_2_	−0.08	(0.67)	−0.19	(1.14)
	*C*_3_	0.57	(0.56)	0.39	(1.17)
	*C*_4_	1.35	(1.54)	1.07	(0.98)
	*C*_5_	1.90	(1.47)	1.74	(1.16)
DPSD	*R*	0.22	(0.20)	0.22	(0.19)
	*d′*	0.55	(0.45)	0.44	(0.40)
	*C*_1_	−1.03	(1.00)	−0.99	(0.96)
	*C*_2_	−0.09	(0.65)	−0.12	(0.80)
	*C*_3_	0.54	(0.54)	0.45	(0.80)
	*C*_4_	1.21	(1.06)	1.11	(1.26)
	*C*_5_	2.41	(2.02)	2.03	(1.67)
MSD	λ	0.60	(0.28)	0.65	(0.27)
	*d*_A_	2.44	(1.68)	1.79	(1.12)
	*C*_1_	−1.07	(1.02)	−1.02	(0.94)
	*C*_2_	−0.04	(0.60)	−0.08	(0.73)
	*C*_3_	0.57	(0.59)	0.54	(0.70)
	*C*_4_	1.26	(1.31)	1.12	(1.01)
	*C*_5_	2.01	(1.42)	1.89	(1.17)

UVSD: unequal variance signal-detection; DPSD: dual process signal-detection; MSD: mixture signal-detection.

#### Comparison of fits

As in the previous experiments, GOF comparisons were performed at individual (see Table 9) and aggregate (see Table 10) levels. The DPSD model gave the greatest percentage of best fits to individual participant’s data in the low-variance condition, with the UVSD coming a close second, and the MSD third. In the high-variance condition, the UVSD and DPSD models provided the joint greatest percentages of best fits, with MSD again following in succession. When fitted to aggregated data from the low-variance condition, the UVSD model provided the best fit, followed by the MSD and DPSD models; the DPSD model fit was rejected on a *G*^2^ significance level of .05. When fitted to aggregated data from the high-variance condition, the DPSD model gave the best fit, followed by the MSD and UVSD models. However, all of these fits were rejected. With no clear GOF hierarchy emerging, these model comparison results are inconclusive, as in previous experiments.

**Table 9.** Goodness of model fits to individual participant’s data in Experiment 3, assessed by *G*^2^.

Condition	Model	Sum of *G*^2^	Percentage of best fits	Percentage of rejected fits
Low variance				
	UVSD	156.22	37.5	7.5
	DPSD	151.70	40	5
	MSD	154.73	22.5	2.5
High variance				
	UVSD	176.07	35	5
	DPSD	171.69	35	7.5
	MSD	167.55	30	7.5

UVSD: unequal variance signal-detection; DPSD: dual process signal-detection; MSD: mixture signal-detection.

Fits rejected if *p* < .05.

**Table 10.** Goodness of model fits to aggregate data in fixed and variable duration conditions (Experiment 3), assessed by *G*^2^.

Condition	Model	Order of best fit	*G* ^2^	*p* value
Low variance				
	UVSD	1	1.25	.87
	DPSD	3	14.48	<.01*
	MSD	2	2.21	.70
High variance				
	UVSD	3	13.28	<.01*
	DPSD	1	10.32	.04*
	MSD	2	12.54	.01*

UVSD: unequal variance signal-detection; DPSD: dual process signal-detection; MSD: mixture signal-detection.

* *p* < .05.

#### Inter-parameter correlations

Following on from our unplanned analyses in Experiment 2, we conducted the same Pearson correlation analyses on parameter estimates in each model in Experiment 3. In the low-variance condition, there was a strong, significant correlation between values of *d* and σ_o_, *r*(38) = .84, *p* < .01, 95% CI [0.71, 0.91]. A moderate positive correlation also emerged between these parameters in the high-variance condition, *r*(38) = .42, *p* < .01, 95% CI [0.12, 0.65]. In the DPSD model, there was no significant correlation between *d*′ and *R* in the low-variance condition, *r*(38) = .16, *p* = .31, 95% CI [−0.16, 0.45]. Despite this, these parameters showed a significant positive correlation in the high-variance condition, *r*(38) = .36, *p* = .02, 95% CI [0.05, 0.60]. MSD model parameters *d*_A_ and λ were significantly negatively correlated in the low-variance condition, *r*(34) = −.57, *p* < .01, 95% CI [−0.75, −0.29], and in the high-variance condition, *r*(34) = −.58, *p* < .01, 95% CI [−0.76, −0.30].

### Discussion

There were no significant differences in σ_o_ between the high- and low-variance conditions, indicating that the UVSD model predicted no change in old item variance when fit to the data. Similarly, *d* did not significantly differ across conditions, indicating an equivalence in memory strength. These predictions were echoed by the DPSD model, in which neither *R* nor *d*′ differed significantly across conditions. In the MSD model, λ remained unchanged while *d*_A_ estimates were greater in the low-variance condition (although the Bayes Factor for this evidence indicated inconclusive evidence). This is indicative of greater memory strength and old item variance in this condition, which, if anything, is not in the direction predicted by the encoding variability hypothesis. Therefore, on the basis of these results, there is no evidence for the encoding variability hypothesis in Experiment 3. It should be noted that this lack of evidence reflects the strength of the correlation between word frequency and recognition confidence. This correlation was significant and negative (mean *r* = −.10 across participants), which was expected given the previously reported negative function of word frequency against memory strength (Gorman, 1961). However, it is possible that realistically, the variance accounted for in this correlation was too small to affect σ_o_, mirroring the lack of a sizable effect of study duration and trial-to-trial attention in Experiments 1 and 2. Once again, despite our best efforts to create a high-variance manipulation of the encoding variable at hand, no such increase in the variance of old item memory strength occurred as a result.

The present model comparison results are also inconclusive, placing the UVSD and DPSD models in close competition as the best model to fit both individual and aggregated data. This comes in contrast with the results of Experiment 2, in which the MSD model was comparatively better than both the UVSD and DPSD models. That these GOF results were not conclusive, both within Experiment 3 and in comparison with Experiment 2, is not surprising given that all three models have been reported to provide fits of similar quality (Yonelinas & Parks, 2007). The present results can serve to reinforce the conclusion that in the future, it would perhaps be more beneficial to test differential predictions of competing models, rather than exclusively assessing their GOF.

Although there were difficulties in testing the encoding variability hypothesis and discriminating between models, it is clear that there is a common trend in the relationship between strength and old item variance. As in Experiments 1 and 2, inter-parameter correlations in the UVSD model showed that the model’s measure of memory strength increased along with old item variance in a strong relationship. The DPSD model’s parameters were also positively correlated, albeit not significantly in the low-variance condition. As previously stated, it is difficult to assess the implications of these correlations upon the relationship between memory strength and old item variance, due to the loosely defined nature of the recollected item distribution. However, the present correlations are similar to those observed in Experiments 1 and 2, indicating a common weak to moderate positive relationship between the parameters. Inter-parameter correlations in the MSD model also echoed previously observed trends, showing a strong negative relationship which predicts simultaneous increases in memory strength and old item variance. These results help to reinforce the positive association between memory strength and old item variance.

## General discussion

Despite the UVSD model’s decades-long prominence as a signal-detection model of recognition memory, the psychological explanation for the old item variance effect has yet to be verified. The encoding variability hypothesis has been proposed as one explanation for the effect (Jang et al., 2012; Wixted, 2007); however, despite its intuitive appeal, we failed to provide confirmation of it in our study. The results of Experiment 1 show that varying study duration from trial to trial had no effect on old item strength variance, compared with when study duration was fixed. In Experiment 2, we found no evidence that varying attention from trial to trial affected old item variance, compared with when attention was relatively constant across trials. Instead, increasing variability in attention actually led to a decrease in old item variance, although Bayes Factors suggested that evidence for this effect, though significant, was inconclusive. In Experiment 3, word frequency did not affect old item variance. Under the encoding variability hypothesis, we expected old item variance to be greater in each variable condition, which it was not. Although our manipulations do not provide evidence against the hypothesis, they demonstrate that, if the hypothesis is indeed correct, it is surprisingly hard to influence old item variance in line with its predictions.

Instead, old item variance tended to be linked to overall strength, such that variance tended to increase with overall strength. The existence of such a link seems intuitive: if the average signal strength for a set of old items is greater, then those items can take on a potentially broader range of strength values, thus increasing variance. In this way, as old item strength is assumed to be greater than new item strength, the variance of the old item distribution will tend to be greater than that of the new item distribution. In Experiments 1 and 3, this was the case; however, as overall memory strength did not differ between each condition, neither did old item variance. In Experiment 2, estimates of memory strength were predicted to be greater by each model in the fixed condition, along with old item variance. Although the encoding variability hypothesis could not account for this effect, when given context by an increase in strength, it can be explained. Despite an initial generalisation that the slope of the *z-*ROC (signifying the ratio of new/old item variance) was unaffected by memory strength (Ratcliff et al., 1992), later evidence has contested this claim based on slopes changing with accuracy manipulations in both previous and new experiments (Glanzer et al., 1999). On balance, these and our findings are consistent with the idea that old item variance is usually linked to the overall level of memory strength, rather than encoding variability per se.

It is important to note here that we assumed that any additional variance created by our manipulations was Gaussian in form. Yet, despite this assumption being reflected in our methods, there is no way of conclusively confirming this due to memory strength’s nature as a latent variable (Rouder et al., 2010). Considering this in Experiment 1, we chose our manipulation of study duration as it would not obviously create a mixture distribution (a view supported by Jang et al., 2012). Our methods in Experiment 2 and Experiment 3 also do not create obvious mixture distributions. Moreover, if our addition of variance was not Gaussian, it is still possible that the old item distribution retains a Gaussian form due to the central limit theorem. This states that even the sum of independent random non-Gaussian variables will be Gaussian in form as the number of such variables increases. When applied to the present experiments, even if either manipulated encoding variable was non-Gaussian, the large number of other potential encoding variables might be expected to push the added strength distribution towards a Gaussian form. For this reason, the assumption that the total old item strength is Gaussian is made, which also allows for the derivation of otherwise computationally difficult or impossible mathematical results (Wixted & Mickes, 2010).

It should also be salient that our failure to provide supporting evidence for the encoding variability hypothesis should not be taken as support for a model of recognition memory where incremental strength added at study does not vary. Indeed, based on the aggregated effects of many factors that affect memory strength during study, the addition of variable strength to old items is plausible. However, it is also possible that the variance contributed by these aggregated factors is not the primary causal contributor to the old item variance effect. In this case, variable strengths could be added to old items at encoding, but this alone may not result in observable effects upon encoding variability. Other possible explanations of the old item variance effect (such as a strength scaling account) are not incompatible with the idea that variable increments of strength are added at study; instead, they dispute the idea that this added variance is the sole cause of the old item variance effect.

Based on our attempts, the difficulty in manipulating a potential encoding variable enough to cause a substantial effect on recognition confidence is clear. Indeed, in the present experiments, no single encoding variable was able to account for a large proportion of added old item variance. We cannot rule out the possibility that there were small effects of our manipulations on old item variance, which we did not have sufficient power to detect. However, a very large (and likely impractical) number of participants would be required to detect such effects. Although this result is unfortunate, the encoding variability hypothesis posits an overall increase in old item variance as a result of the compounded effect of many different variables. Therefore, if manipulating a single encoding variable fails to increase old item variance, compounding the effects of multiple encoding variables in a single study phase may add old item variance successfully. Based on our results, it seems that a new experimental approach based upon this concept could provide a better chance of finding a strong test of encoding variability. Further research could explore this possibility, although this would require many theoretical and methodological considerations to implement experimentally.

It is possible that encoding variability could still affect old item variance by manifesting itself in a way that has yet to be tested. However, irrespective of methodology, there are several theoretical considerations which work against efforts to test the hypothesis. First, despite the specific mathematical assumptions made by the hypothesis, the definition of an encoding variable as any factor affecting memory strength remains broad and could encompass a wide range of variables and processes. For example, attention could be affected not only by simultaneous task demands as in Experiment 2, but by other cognitive factors or a variety of sensory distractions. Although this presents many possibilities for further study, the task of exhaustively testing every possible encoding variable to determine its effect (if any) on old item variance quickly becomes a difficult challenge. Therefore, the encoding variability hypothesis becomes difficult to falsify in its current conceptualisation.

Second, some of these possible encoding variables, such as the previous example of cognitive factors in attention, are difficult to experimentally manipulate due to the distributional assumptions of the hypothesis. In order to avoid the inclusion of mixture distributions as Koen and Yonelinas (2010) encountered, normally distributed variability has to be added experimentally across a study phase, as we attempted in our methods. Indeed, even if additional variance is non-Gaussian, it is still essential that this variance is added across a study phase so as not to confound any other given distributional assumption through mixture. There are a multitude of variables which have been shown to affect memory strength, but may be problematic to manipulate in this way; plausible examples could include emotion regulation (Richards & Gross, 2000), emotional content of stimuli (McCloskey et al., 1988), the “bizarreness” of stimuli (McDaniel et al., 1995), and many likely others. This would make the already difficult task of exhaustively testing the encoding variability hypothesis even more challenging.

Third, in any recognition memory experiment, it can be assumed that the baseline variability of memory strength is already quite high. Regardless of the method, attention to the task at hand is likely to show some fluctuation throughout a study phase, as previously discussed in Experiment 1. The time between the presentation of each stimulus at study and at test will vary as well, which may also add variation in memory strength. The memorability of stimuli will also vary according to a multitude of factors. Based on each of these points, any further experimentally added variance would have to be very strong to have a significant increase on the total old item variance, on top of the baseline amount.

Finally, it is reasonable to assume that the correlation between the baseline memory strength of a stimulus and the strength added to it during study is negative. In methods that work to experimentally increase old item variance, the variance of the old item distribution in the UVSD model can be expressed as

(7) $$\sigma_{o}^{2}=\sigma_{B}^{2}+\sigma_{Y}^{2}+2\rho \sigma_{Y}\sigma_{B}$$

where *B* and *Y* represent the baseline and added strength distributions as defined in Equations 3 and 4, and ρ is the correlation between baseline and added strength. A negative value of ρ in this equation therefore works against any attempt to add variance with an experimental manipulation. This possibility has been previously considered by Jang et al. (2012), who stated that the encoding variability hypothesis assumes that any such negative correlation is too small to counteract attempts to add variance. Whether this is the case is debatable, although it holds that added variance is mitigated to some degree even in the case of a small negative correlation. This issue, compounded by the other practical and theoretical shortcomings, limits the testability of the encoding variability hypothesis.

Even though no experimental evidence of the encoding variability hypothesis exists as of yet, this alone does not damage the UVSD model’s legitimacy. As the model does not depend upon the encoding variability hypothesis being correct, a lack of evidence for this hypothesis does not impair its functionality. It is also still possible that added strength at encoding varies in some way. The results of this study do, however, present a challenge for proponents of the encoding variability hypothesis—to find a set of circumstances in which a valid method can lead to an increase in old item variance, as a result of encoding variability. Alternatively, proponents could reconceptualise the encoding variability hypothesis (or suggest a new explanation altogether) to explain the results of the present study and previous work that finds no evidence for its current conceptualisation (Koen et al., 2013). It has been speculated that a separate process during the retention interval between study and test is responsible for mitigating or reversing the effects of encoding variability (Koen et al., 2013); however, this has not been tested. Any unique predictions relating to old item variance made by the DPSD or MSD models could also be identified and tested further. Equally, the suggestion of an association between strength and variance could provide an alternative explanation, though again, further research is needed to fully evaluate this claim.

To conclude, we were unable to find evidence for the encoding variability hypothesis in any of our experiments as a result of manipulating study duration (Experiment 1), attention through simultaneous task demands (Experiment 2), or word frequency (Experiment 3). In fact, in Experiment 2, old item variance was predicted to be greater as a result of encoding variability in the variable condition, but it was actually significantly greater in the fixed condition, along with memory strength. Inter-parameter correlations in each experiment also supported a positive relationship between old item variance and memory strength. These results are compounded by the inherent difficulty in testing the hypothesis, from both experimental and theoretical perspectives. Thus, future efforts could use new methods to test the encoding variability hypothesis, or suggest a new explanation for the old item variance effect in the UVSD model. The link between memory strength and old item variance could also be explored further as an explanation.

## Supplemental Material

QJE-STD-19-192.R2-Supplementary_Materials – Supplemental material for The unequal variance signal-detection model of recognition memory: Investigating the encoding variability hypothesis Click here for additional data file. Supplemental material, QJE-STD-19-192.R2-Supplementary_Materials for The unequal variance signal-detection model of recognition memory: Investigating the encoding variability hypothesis by Rory W Spanton and Christopher J Berry in Quarterly Journal of Experimental Psychology

## Notes

1. MATLAB programs used to run this, and each other experiment in this article are available on our OSF project page.

2. In our preregistration, we stated that statistical indices *d′* and *C* would be calculated to measure recognition accuracy and bias, respectively, on the basis that previous researchers had also reported them. However, these indices assume that new and old item variances are equal. As this assumption has been conclusively rejected by the UVSD model’s superiority over an equal variance signal-detection model, hit and false-alarm rates were instead used to provide a broad assessment of recognition performance before fitting each model.

3. Estimates of σ_o_ calculated from *z-*ROC slopes (derived from aggregated data) and fits to aggregated data supported this conclusion. The value of σ_o_ calculated from *z-*ROC slopes was marginally lower in the variable condition (σ_o_ = 1.28) than in the fixed condition (σ_o_ = 1.35). When models were fitted to aggregate data, the same trend emerged; the UVSD model predicted slightly lower old item variance in the variable duration condition (σ_o_ = 1.29) than in the fixed duration condition (σ_o_ = 1.36).

4. We acknowledge that the manipulation which we present in Experiment 2 differs from a traditional *n*-back task. As the “odd or even” decision is the same in each trial, it is possible for a participant to hold only the response associated with the stimulus in memory to the complete the task and not the stimulus itself. In contrast, the identity of the stimulus itself is required to perform the associated response in a standard *n*-back task. However, as the participant still has to retain some information about a target stimulus (despite this not necessarily including the numeric identity of the stimulus) from a preceding trial, we henceforth refer to our experimental manipulation as an “*n*-back task.”
