# Supplementary material for: The unequal variance signal-detection model of recognition memory: Investigating the encoding variability hypothesis
Source: Q J Exp Psychol (Hove). 2020 Feb 27;73(8):1242–60. doi: 10.1177/1747021820906117 (PMC7338698; doi:10.1177/1747021820906117)

Supplementary Material for:

**The Unequal Variance Signal-Detection Model of Recognition Memory: Investigating the Encoding Variability Hypothesis**

Rory W. Spanton & Christopher J. Berry

**Supplementary Material A: Parameter Recovery Simulations**

It is important to determine whether the parameter values of the UVSD model can be successfully recovered from data that is simulated from the model when a range of values of σ_O_ have been used to simulate the data. To investigate this, we conducted parameter recovery simulations. Five data sets, each consisting of data from 40 simulated participants, were generated from the UVSD model; *d* was fixed at 1.1. For each simulated ‘participant’, each criterion (*C*_1_ to *C*_5_) was sampled from a truncated normal distribution with upper and lower bounds *a* and *b*, so that

$a<C_{i} \sim N\left( \mu_{C_{i}}, \sigma_{C_{i}} \right)<b$ [A1]

where *a* > *C_i_*_-1_ and *b* < *C_i_*_+1_. This ensured that the correct ordering of the criteria was preserved (i.e., *C*_1_, *C*_2_, *C*_3_, *C*_4_, *C*_5_). The constraints upon each of these distributions are reported in Table A1 below.

Table A1.

*Generative constraints upon criteria in the UVSD model.*

| Parameter | µ | σ | *a* | *b* |
| --- | --- | --- | --- | --- |
|  |  |  |  |  |
| *C*_1_ | -0.2 | 0.1 | -0.35 | -0.15 |
| *C*_2_ | 0.2 | 0.1 | 0.05 | 0.35 |
| *C*_3_ | 0.6 | 0.1 | 0.45 | 0.75 |
| *C*_4_ | 1.0 | 0.1 | 0.85 | 1.15 |
| *C*_5_ | 1.4 | 0.1 | 1.25 | 1.55 |

In each data set, σ_o_ was set to 1, 1.25, 1.5, 1.75 and 2 respectively to simulate different levels of old item variance. For each simulated participant, responses to 260 simulated trials (130 old/new) were generated, and each model was then fitted to their data. Mean estimated values of σ_o_ and *d* were directly compared with the true values of σ_o_ and *d* from each dataset (see Table A2). The DPSD and MSD models were also fit to the data generated by the UVSD model in order to investigate how these models would adapt their parameters under these conditions; *d′*, *R* and *C*_(1,… 5)_ were estimated by the DPSD model, and *d*_A_, λ and *C*_(1,… 5)_ were estimated by the MSD model. Mean *z*-ROC slopes were also calculated for model fits to each generated data set as an independent measure of old item variance.

Table A2.

*Strength and old item variance parameter estimates and mean z-ROC slopes for the UVSD, DPSD and MSD models, in comparison to true values of σ_o_ used to simulate data from the UVSD model.*

| Model and Estimated Parameter | | True σ_o_ | | | | |
| --- | --- | --- | --- | --- | --- | --- |
|  |  | 1 | 1.25 | 1.5 | 1.75 | 2 |
| UVSD |  |  |  |  |  |  |
|  | σ_o_ | 1.03 | 1.26 | 1.59 | 1.77 | 2.02 |
|  | *d* | 1.12 | 1.15 | 1.12 | 1.15 | 1.11 |
|  | Mean slope | 0.99 | 0.81 | 0.65 | 0.58 | 0.51 |
| DPSD | |  |  |  |  |  |
|  | *R* | 0.07 | 0.19 | 0.31 | 0.34 | 0.37 |
|  | *d'* | 1.02 | 0.77 | 0.42 | 0.30 | 0.14 |
|  | Mean slope | 0.95 | 0.83 | 0.68 | 0.63 | 0.55 |
| MSD |  |  |  |  |  |  |
|  | $\lambda$ | 0.93 | 0.75 | 0.56 | 0.52 | 0.46 |
|  | *d*_A_ | 1.22 | 1.53 | 2.05 | 2.49 | 4.14 |
|  | Mean slope | 0.94 | 0.80 | 0.65 | 0.61 | 0.55 |

The differences between true and estimated values of σ_o_ were small while *d* remained around a constant value, confirming that the parameters of the UVSD model can indeed be recovered at different levels of old item variance. As expected, the DPSD model’s estimated value of *R* was predicted close to 0 when the true value of σ_o_ = 1, and increased with this true value. Estimates of *d′* decreased as old item variance became greater. Since *R* affects both the mean strength and variance of old items, increases in *R* to accommodate greater variance would also increase the expected mean strength of old items (which did not vary in the simulated data, i.e., because true *d* = 1.1). Therefore, *d′* decreasing compensated for any implied increases in memory strength as a result of *R* increasing, while simultaneously increasing the variance of the old item distribution further. In this way, the DPSD model attempts to mimic greater old item variance in the UVSD model by increasing *R* and lowering *d′*. The MSD model’s estimated value of λ was close to 1 when the true value of σ_o_ was equal to 1, and decreased to approximately 0.5 as the true value of σ_o_ increased, whereas *d*_A_ increased with the true value of σ_o_. Since a decrease in λ signifies a decrease in mean strength, the increase in estimates of *d*_A_ compensates for this effect by increasing mean strength, as well as further increasing old item variance. In this way, an interaction between λ and *d*_A_ can account for increases in old item variance while balancing overall strength.

In each model, parameter estimates for higher old item variance conditions are different from those in lower variance conditions, with unique interactions between each pair of parameters characterising each model’s fit to data. However, the mean z-ROC slopes calculated from each model fit are similar at every level of generative old item variance. This means that increases in encoding variability are unlikely to result in any of the models making distinguishable predictions about the slope of the *z*-ROC. Regardless, each model’s parametric behaviour and subsequent predictions are clearly identifiable, at least within the parameter space explored here.

**Supplementary Material B: Model Recovery Analysis**

In situations where models are compared, various GOF statistics can be used to assess the quality of model fits. Although these are useful for comparing models, it is still important to determine whether a model can provide a better fit than its competitors can if it had generated the data, according to a particular statistic. Consider the following example; in a case where Model A generates a data set, another model (B) may give a better fit statistic (e.g., lower *G*^2^) when fitted to that data. This is because Model B is able to mimic other models, given that it can account for data generated using parameters and assumptions that differ from its own. A model with this behaviour would not be able to make informative predictions about the true nature of a process due to the wide range of data types it accommodates. However, based on the test statistic alone, Model B would be incorrectly selected as the true model, contrary to the fact that Model A generated the data.

As well as the models themselves, the number of trials comprising the data which the model is fitted to should also be considered. Although it is more likely that true generative models can be recovered by a GOF statistic at higher trial sizes, this may not occur at lower trial sizes. However, if it is possible to consistently recover true models at a higher trial size, then those models are not over flexible; it is instead the case that the GOF statistic being used can only identify a true model at higher trial sizes, due to a reduction in sampling error. Therefore, it is important to conduct model recovery simulations to investigate both the mimicry of the models in question, but also the ability of a chosen GOF statistic to distinguish a true generative model. Previous research has assessed the flexibility of the UVSD, DPSD and MSD models using yes/no and two alternative forced choice methods (Jang, Wixted, & Huber, 2009; Kellen et al., 2013). While this research found that all types of true model were able to be recovered, and that the UVSD model provided the best fit to data, we are unaware of any investigation that has compared mimicry of the UVSD, DPSD and MSD models when fitted to 1-6 confidence rating data. To investigate this, we conducted model recovery simulations.

In our model recovery simulations, three datasets were generated using parameters from the UVSD, DPSD, and MSD models respectively. Values of *R* and λ were sampled from two uniform distributions, so that

$R \sim U\left( a=0, b=1 \right)$ [B1],

$\lambda\sim U\left( a=0, b=1 \right)$ [B2].

For every model, each other parameter was sampled from a truncated normal distribution which exists between upper and lower bounds, *a* and *b*, so that

$a< X \sim N\left( \mu_{X}, \sigma_{X} \right)<b$ [B3]

where *X* is the parameter in question. The constraints upon these parameters are reported in Table B1 below.

Table B1.

*Generative constraints upon parameters in the UVSD, DPSD, and MSD models.*

| Model | Parameter | µ | σ | *a* | *b* |
| --- | --- | --- | --- | --- | --- |
| UVSD |  |  |  |  |  |
|  | σ_o_ | 1.3 | 0.2 | 1.0 | ∞ |
|  | *d* | 1.1 | 0.2 | 0.8 | 2.3 |
| DPSD |  |  |  |  |  |
|  | *d′* | 1.5 | 0.2 | 0.8 | 2.3 |
| MSD |  |  |  |  |  |
|  | *d*_A_ | 1.8 | 0.2 | 0.8 | 2.3 |
| All |  |  |  |  |  |
|  | *C*_1_ | -0.2 | 0.1 | -0.4 | 0 |
|  | *C*_2_ | 0.2 | 0.1 | 0.1 | 0.3 |
|  | *C*_3_ | 0.4 | 0.1 | 0.3 | 0.5 |
|  | *C*_4_ | 1.2 | 0.1 | 0.9 | 1.5 |
|  | *C*_5_ | 2.0 | 0.1 | 1.6 | 2.4 |

In each dataset, responses from 100 virtual ‘participants’ were simulated. The values of the free parameters of each model were then estimated for every ‘participant’, accounting for all nine possible data type x model fit combinations. This analysis was performed in cases where the total number of simulated test trials was equal to 260, 1000, 5200, and 20000 for each participant. As 260 trials (130 old, 130 new) are used in each condition of Experiments 1 and 2, the results of this simulation are applicable to participant level GOF analyses. 5200 is the total number of test trials across participants in the main experiment (130 trials × 40 participants), and so this case applies to assessing GOF to aggregated data. To gain a greater understanding of the patterns of flexibility of each model, data with 1000 trials was generated to provide a trial number between 260 and 5200, and the 20000 trial data was simulated to represent a theoretical case with a very high number of trials. The model fits from each combination of data type, model fitted and number of trials were analysed using *G*^2^ (see below).

Table B2.

*Percentages of Best Fit Using G^2^ at trial sizes 260, 1000, 5200, and 20000.*

| Data Type and Model Fitted | | Number of test trials | | | |
| --- | --- | --- | --- | --- | --- |
|  |  | 260 | 1000 | 5200 | 20000 |
| UVSD | |  |  |  |  |
|  | UVSD | 55% | 71% | 83% | 90% |
|  | DPSD | 11% | 10% | 3% | 1% |
|  | MSD | 34% | 19% | 14% | 9% |
| DPSD | |  |  |  |  |
|  | UVSD | 14% | 7% | 1% | 1% |
|  | DPSD | 57% | 87% | 95% | 98% |
|  | MSD | 29% | 6% | 4% | 1% |
| MSD |  |  |  |  |  |
|  | UVSD | 32% | 19% | 17% | 24% |
|  | DPSD | 23% | 28% | 32% | 26% |
|  | MSD | 45% | 53% | 51% | 50% |

*Note.* The values correspond to the percentage of cases (out of 100) in which the model had the smallest *G^2^* value. UVSD = unequal variance signal-detection model; DPSD = dual process signal-detection model; MSD = mixture signal-detection model.

At all numbers of test trials, each model was recovered in the majority of cases. This was true even when the number of trials was relatively low, although the rate at which the true model was recovered was, generally, clearly lower than when the number of trials was relatively high. However, because each model accounted for the highest percentage of best fits to its own generated data at any number of trials tested, none of them show problematic mimicry in either individual or aggregate GOF analyses.

**Supplementary Material C: Model Fitting Procedure**

To fit the models, we wrote custom R scripts that returned the maximum-likelihood estimation (MLE) parameter values, given starting values for the parameters of each model. To derive the starting values, we first calculated initial (i.e., rough) estimates of the parameters, as follows. The response criteria were estimated with respect to the new item distribution. The lowest criterion was estimated with the equation

$\hat{C}_{1}= z\left[ 1-\left( \frac{\sum X_{2|N}}{n} \right) \right]$ [C1]

where *z* is the inverse of the cumulative normal distribution function, *X*_2_*_|N_* is the sum of all 2 (i.e., “Probably New”) recognition ratings or higher to new items, and *n* is the total number of new items. To ensure that each subsequent criterion would be greater than the previous one, the MLE procedure estimated the differences between subsequent criteria, with the initial values estimated as

${d(\hat{C}}_{i})= z\left[ 1-\left( \frac{\sum X_{i+1|N}}{n} \right) \right]-z\left[ 1-\left( \frac{\sum X_{i|N}}{n} \right) \right]$ [C2]

where *i* represents the criterion number in question. For the initial estimates of the distance parameters in each model, we used *dʹ*, computed from the data as

$\hat{d}ʹ= z\left[ P\left( H \right) \right]-z[P(FA)]$ [C3]

where *P*(*H*) is the hit rate and *P*(*FA*) is the false alarm rate. In the UVSD model, the initial estimate of σ_o_ was equal to 1 in Experiments 1 and 2. In Experiment 3, it was derived as

$\hat{\sigma_{o}}= \frac{1}{z-ROC slope}$ [C4].

We made this change to give an initial estimate that reflects the UVSD model’s assumption of unequal variance. We assumed that, given that starting values in our fit routine are sampled with variance from initial estimates (see below), this change would not noticeably increase the overall accuracy of the UVSD model in Experiment 3.

The initial parameter estimates were then used to determine the starting values for each parameter. In each model, starting criterion values were sampled in the same way. The starting value of *C*_1_ was sampled from a normal distribution with mean equal to *Ĉ*_1_, and standard deviation equal to 0.2. Likewise, the starting value of each criterion difference was given as:

$d(C_{i})\sim N(\mu= {d(\hat{C}}_{i}), \sigma=0.2)$ [C5].

Thus, *C_i_* = *C_i_*_-1_ + *d*(*C*_i_). Distance parameters in each model (*d*, *d′*, and *d*_A_) were also sampled from a normal distribution with a mean of *d̂ʹ* and standard deviation of 0.2. Starting values for σ_o_ were sampled from a normal distribution with a mean of $\hat{\sigma_{o}}$, and standard deviation of 0.2. Starting values for *R* and λ were both sampled from a uniform distribution described by Equations B1 and B2.

**MLE Function**

Estimating the parameter values of the models using MLE involved determining the likelihood of the recognition rating on each trial, given particular parameter values of the models. The parameter values that maximised the summed log-likelihood across all test trials were determined using the Nelder-Mead algorithm as implemented in the optim function of R (for further explanation of MLE methods for the UVSD, DPSD and MSD models, see Dunn, 2010). This procedure of parameter generation and optimisation was repeated 20 times; afterwards, the set of parameter values that returned the greatest log-likelihood was selected as the maximum likelihood estimates. This process was repeated for each participant’s data in either condition, or in the case of the rest of our GOF analyses, aggregated data from each condition.

**Supplementary Material D: Aggregate ROCs**

*Figure D1.* ROC plots of aggregate model fits in Experiment 1 (fixed condition: panel A; variable condition: panel B), Experiment 2 (fixed condition: panel C; variable condition: panel D) and Experiment 3 (low variance condition: panel E; high variance condition: panel F). The area under the curve (AUC) values are shown against the key in each panel.


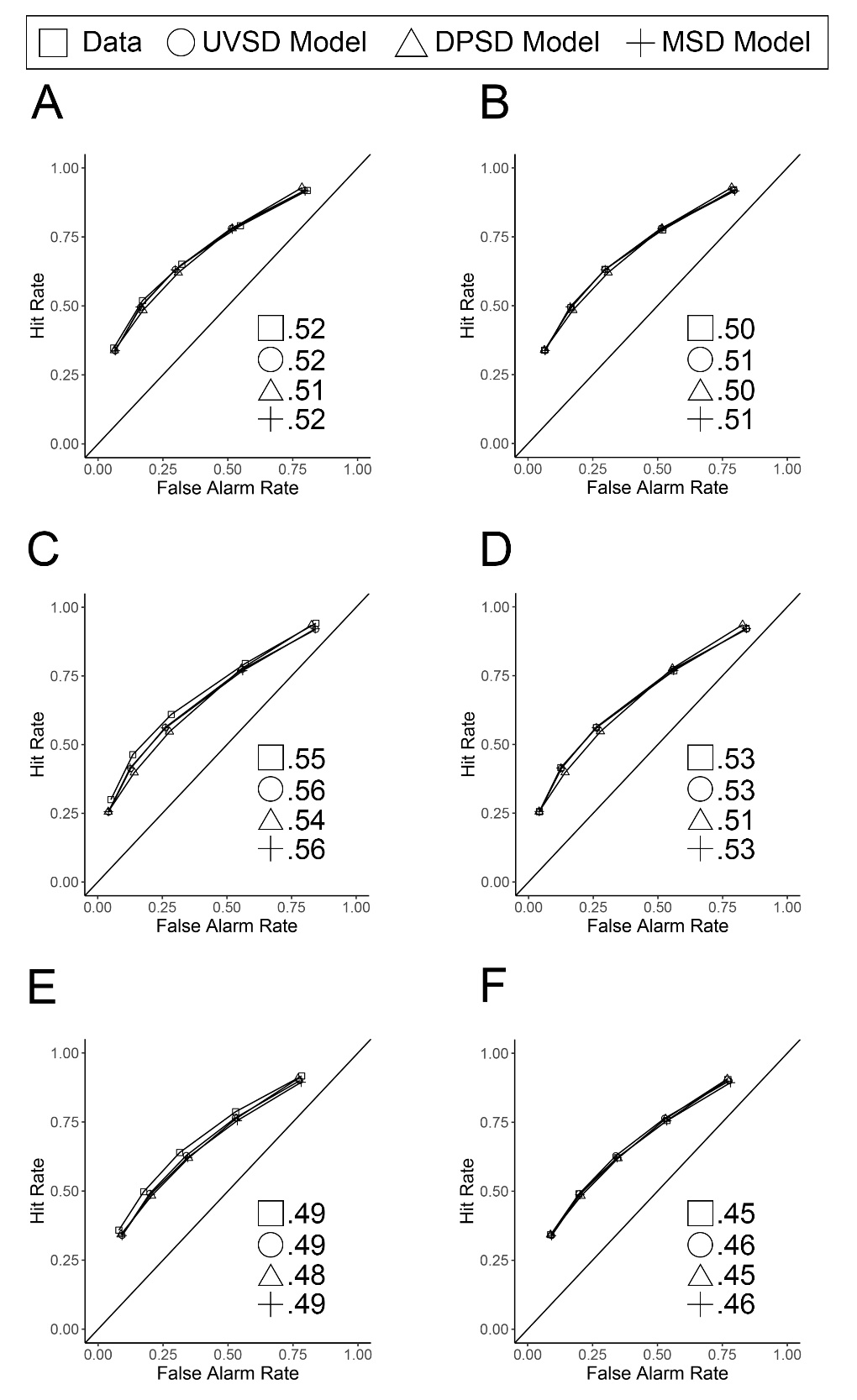


*Figure D2. z-*ROC plots of aggregate model fits in Experiment 1 (fixed condition: panel A; variable condition: panel B), Experiment 2 (fixed condition: panel C; variable condition: panel D) and Experiment 3 (low variance condition: panel E; high variance condition: panel F). *z*-ROC slope values are shown against the key in each panel.


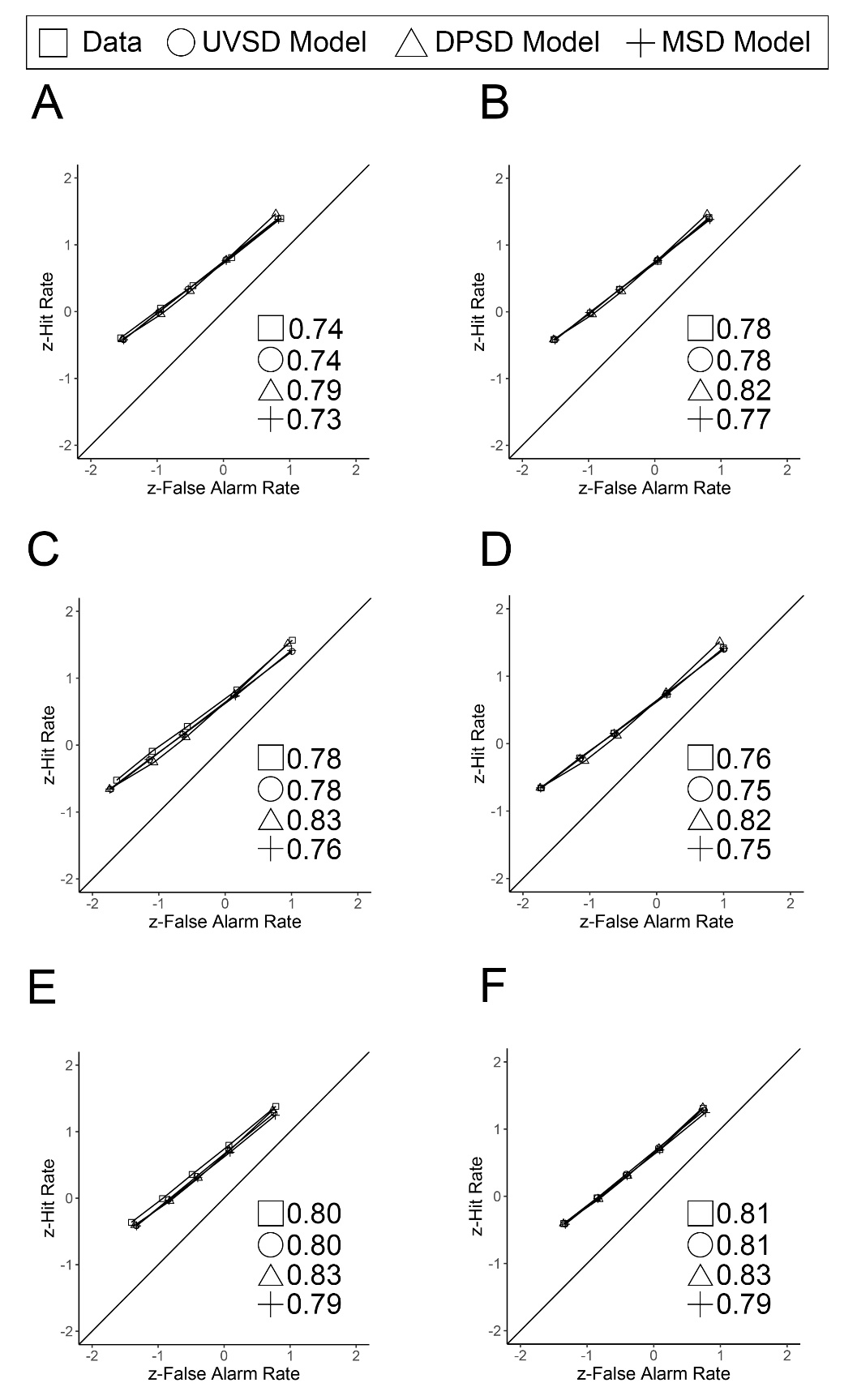

Supplement: QJE-STD-19-192.R2-Supplementary_Materials – Supplemental material for The unequal variance signal-detection model of recognition memory: Investigating the encoding variability hypothesis [file QJE-STD-19-192.R2-Supplementary_Materials.docx]
